# Theoretical Models and Computational Analysis of Action Potential Dispersion for Cardiac Arrhythmia Risk Stratification

**DOI:** 10.3389/fcvm.2021.649489

**Published:** 2021-03-05

**Authors:** Uma Mahesh R. Avula, Lea Melki, Jared S. Kushner, Stephanie Liang, Elaine Y. Wan

**Affiliations:** ^1^Division of Nephrology, University of Mississippi, Jackson, MS, United States; ^2^Division of Cardiology, Department of Medicine, Vagelos College of Physicians and Surgeons, Columbia University, New York, NY, United States; ^3^Department of Medicine, Prince of Wales Hospital, Hong Kong, China

**Keywords:** action potential duration, optical mapping of calcium and action potentials, action potential dispersion, cardiac arrhythmia, heart

## Abstract

Reentrant cardiac arrhythmias such as atrial fibrillation (AF) and ventricular fibrillation (VF) are common cardiac arrhythmias that account for substantial morbidity and mortality throughout the world. However, the mechanisms and optimal ablation treatment strategies for such arrhythmias are still unclear. Using 2D optical mapping of a mouse model with AF and VF, we have identified regional heterogeneity of the action potential duration (APD) in the atria and ventricles of the heart as key drivers for the initiation and persistence of reentry. The purpose of this paper is to discuss theoretical patterns of dispersion, demonstrate patterns of dispersion seen in our mouse model and discuss the computational analysis of APD dispersion patterns. These analyses and discussions may lead to better understanding of dispersion patterns in patients with these arrhythmias, as well as help comprehend whether and how reducing dispersion can lead to arrhythmia risk stratification and treatment strategies for arrhythmias.

## Introduction

Complex reentrant cardiac arrhythmias such as atrial fibrillation (AF), and ventricular fibrillation (VF) are prevalent clinical cardiac diseases affecting millions of people around the world. AF may result in clot formation in the atria of the heart leading to stroke, and when uncontrolled, may lead to heart failure. AF has also been found to be an independent risk factor for cognitive decline and dementia (1). The mechanisms of reentry causing AF are not clear and the treatment options such as pharmaceutical therapy and ablation therapy are only met with moderate success (2, 3).

It is thought that the mechanism of AF reentry in the atria of the heart also occurs in the ventricles, deteriorating into VF (4). VF accounts for >700,000 sudden cardiac deaths in the US and Europe (5). The risk of VF increases in patients after myocardial infarction, but can also occur in patients with genetic abnormalities such as mutations in sodium and calcium channels. This suggests that anatomical and electrophysiological alterations due to genetic or physiological causes may cause a predisposition to reentry (4, 6, 7).

Reentry refers to the persisting activation sequence of a wavefront that repeatedly propagates around an anatomic or functional region, referred to as a core (5). The substrate requirements for reentry include: (i) slow conduction in one limb allowing recovery of excitation just in time for re-excitation by the depolarizing wavefront; (ii) unidirectional conduction block; and (iii) inhomogeneous tissue causing conduction abnormalities, which may be due to an anatomical obstacle, or differences in conduction velocity due to altered electrophysiological properties in the myocardial tissue. Allessie et al. have shown that dispersion of refractory period of 11–16 milliseconds (ms) was sufficient to allow reentry around a line of conduction block of 5 millimeter (mm) after premature stimulation, also known as “short excitable gap reentry” (8, 9).

Increased temporal dispersion of excitation recovery in the heart has previously been established to enhance the development of complex reentry, namely fibrillation and nonuniform recovery of excitability. This further emphasizes the role of nonuniformity of excitability and conduction velocity during the relative refractory period in the initiation of turbulent impulse propagation (9). Dispersion of repolarization and recovery of excitability are also influenced by heart rate and the autonomic nervous system (10). Regional differences in refractoriness or heterogeneity in the passive electrical coupling of the myocardial cells may cause local areas of conduction block and irregular propagation of early premature impulses (9). Areas of longer refractory period may coincide with sites of unidirectional conduction block and cause intramyocardial reentry, e.g., between the endocardium and epicardium (11).

The refractory period is the interval from depolarization to the recovery of excitability (8, 12). Action potential duration (APD) is the time interval from the onset of phase 0 to the recovery of the membrane potential to the resting level. APD can be measured at 50% and up to 90% of repolarization. Over the years, the use of dispersion and heterogeneity has been translated to clinical indicators of repolarization abnormalities, such as QT interval dispersion on the 12-lead electrocardiogram or electrogram dispersion (13, 14). Repolarization time and refractory period have also been measured using a single cell microelectrode, monophasic action potentials (MAPs) (15), and extra stimulus techniques, during electrophysiology study and optical mapping (8).

In this paper, we will present APD dispersion data from a mouse model of atrial and ventricular fibrillation. This double transgenic (TG) mouse model of tetracycline inducible, cardiac-specific overexpression of F1759A SCN5A, initially created to study long QT3, spontaneously develops sustained atrial and ventricular fibrillation, secondary to late persistent sodium current (16). Dispersion will be quantified here by comparing the differences in APD within a tissue, region or area. We will measure the standard deviation (std) of APD across neighboring pixels within a selected area. A low value of dispersion suggests that the data are closely clustered and the tissue is relatively electrophysiologically homogeneous, whereas, a high value of dispersion indicates that there is a clear discrepancy between neighboring data points and an increased potential for reentry.

### Theoretical Models of APD Dispersion

**Figure 1:**
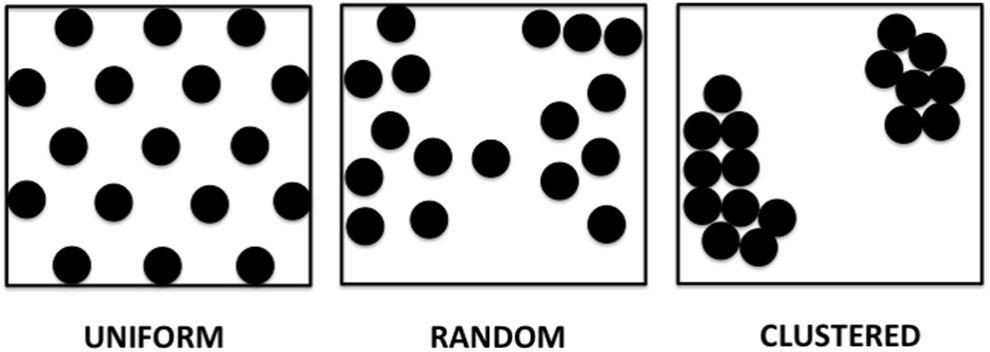
Theoretical models of APD dispersion proposed.

We propose in this paper several patterns of APD dispersion that may be theoretically observed (Figure 1).

- **Uniform dispersion:** areas of dispersion that are evenly spaced over a spatial region of the tissue. This pattern can be observed in genetically modified expression systems, where selected areas have higher dispersion, such as in monolayers with adenovirus inoculation allowing for channelrhodopsin light activation.
- **Random dispersion:** areas of dispersion that have unpredictable distribution. A given area of dispersion is equally likely to occur at any location. There is no cell specific regulation causing dispersion evident in that area, which can occur with either relatively low sodium channel expression or little fibrosis or scar. This can even be present for example, in young mice with a structurally normal heart and little to no fibrosis.
- **Clustered dispersion:** areas of dispersion that are clumped into groups, causing a patchy distribution or islands of areas of dispersion, especially near the anatomic borders, for example, post-myocardial infarction with dispersion along border zones.

## Materials and Methods

### Transgenic Mice

The TG mouse line, F1759A-Na_V_1.5, was generated as previously described (16). Both male and female genders were included, between 3 and 12 months of age. All animal experiments were performed according to NIH guidelines. The Institutional Animal Care and Use Committee at Columbia University approved all animal experiments. Non transgenic littermates were used as controls.

### Optical Mapping Data Acquisition and Processing

Optical mapping of the Langendorff perfused mice hearts was performed (16) using a complementary metal-oxide-semiconductor (CMOS) camera (MICAM Ultima, SciMedia). Movies acquisitions were performed at 1,000 f/s for 4–5 s, with 100 × 100 pixel resolution (0.095 mm per pixel). Image processing was performed using a custom software, PV-WAVE (Precision Visuals - Workstation Analysis and Visualization Environment, Visual Numerics, Inc) (17). Dominant frequency (DF) and phase maps were obtained in AF or ventricular tachycardia (VT)/VF, while APD and conduction velocity maps were obtained with atrial or ventricular pacing at 10-Hz. Pacing-induced AF and VT/VF were assessed by 3 attempts of burst pacing at twice the excitation threshold of the left atrium and left ventricle, respectively (20 Hz, amplitude 0.5–2.0 mA, 5 ms). Two wires in the bath acted as single vector ECG monitoring for the Langendorff perfused hearts. Average APD, maximum APD, and APD dispersion [std(APD)], were calculated for the whole atrium and ventricle, and compared between the regions of highest singularity point density (SPD) to a neighboring 10 × 10 pixel area (17). High APD gradients were defined as regions where the difference between a long APD and a neighboring short APD within a 10 × 10 pixel area was greatest. Dispersion was calculated using a customized processing code in MATLAB.

### Statistical Analysis

Group data are presented as mean ± standard error of the mean (SEM). Statistical comparisons between the groups were tested using a one sample *t*-test. Values of *P* < 0.05 were considered statistically significant. All statistical analyses were performed using Prism 6.0 (San Diego, CA, 2018).

## Results

**Figure 2:**
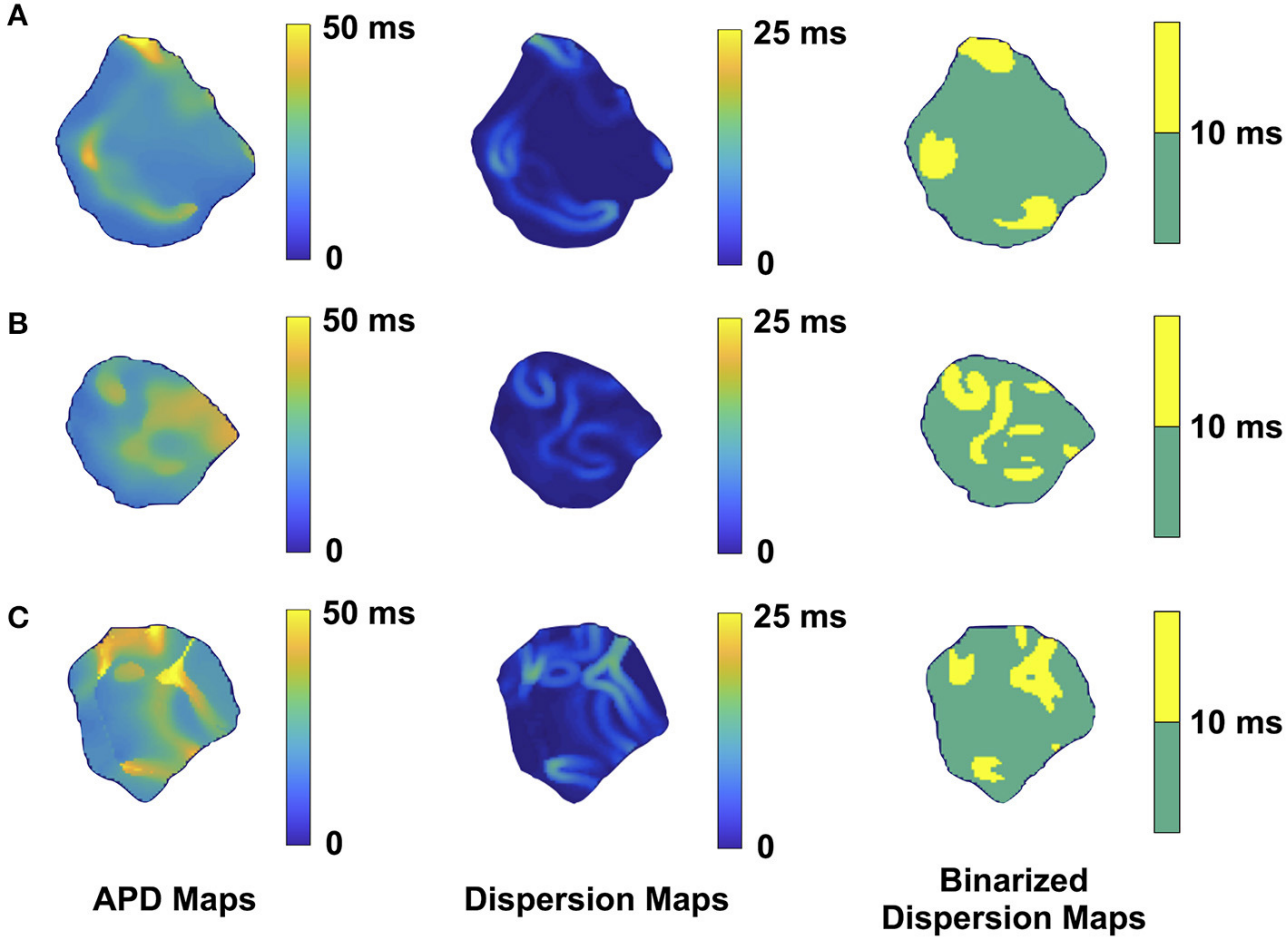
Representative optical mapping images in the atria of three TG mice **(A–C)** with AF. The column on the left shows the APD maps of the epicardial surface of the atria captured during optical mapping. The middle column displays the corresponding APD dispersion maps. The column on the right demonstrates the binary APD dispersion patterns obtained when applying a threshold for areas with APD differences >10 ms in the atria. The mice were mapped after hyperkalemic induced sinus conversion and 10 Hz pacing.

**Figure 3:**
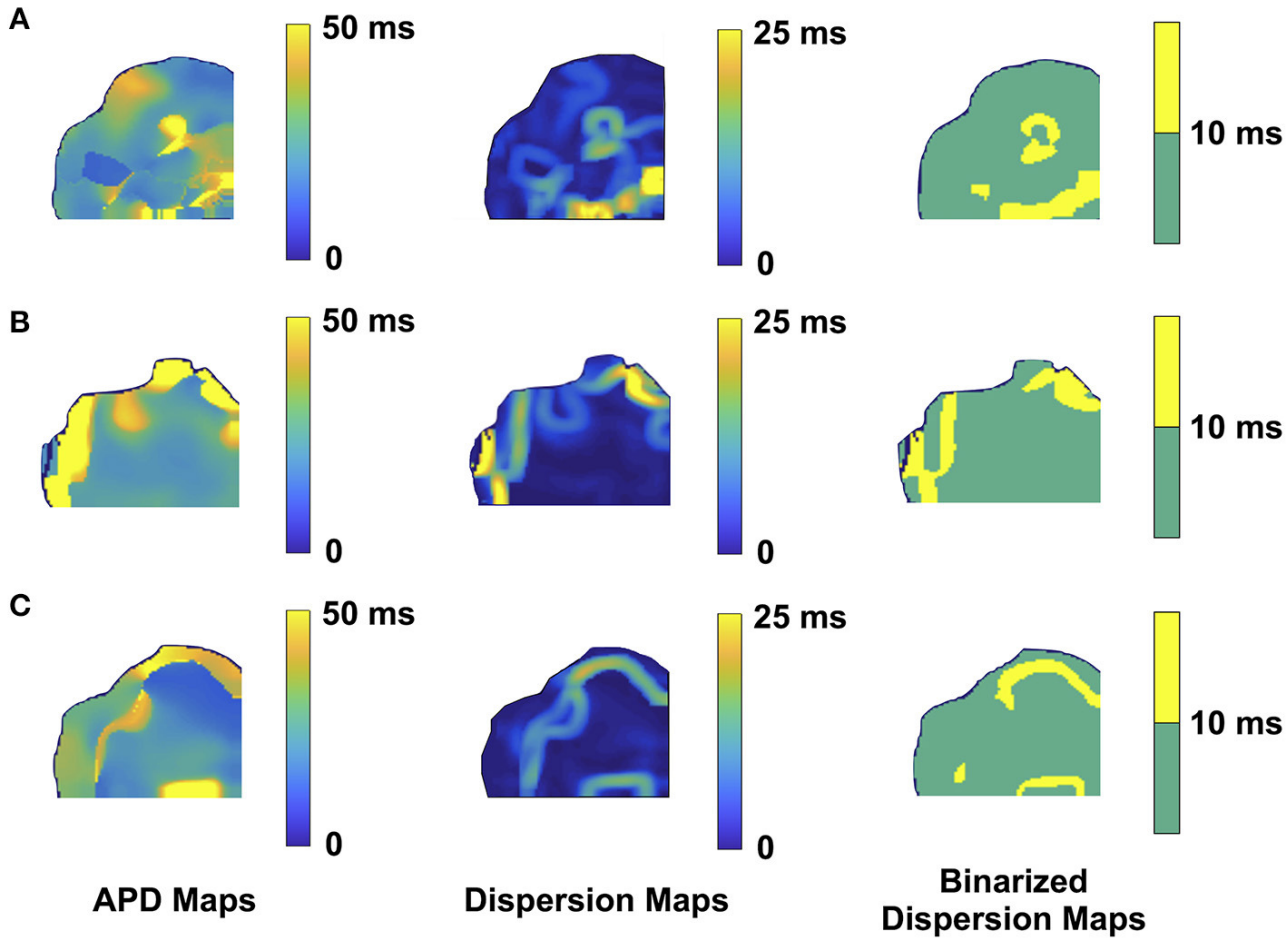
Representative optical mapping images in the ventricles of three TG mice **(A–C)** with VT/VF. The column on the left shows the APD maps of the epicardial surface of the ventricle captured upon optical mapping. The middle column displays the corresponding APD dispersion maps. The column on the right demonstrates the binary APD dispersion patterns obtained when applying a threshold for areas with APD differences >10 ms in the ventricle. The three mice with spontaneous and sustained VT/VF were captured in normal sinus rhythm.

**Figure 4:**
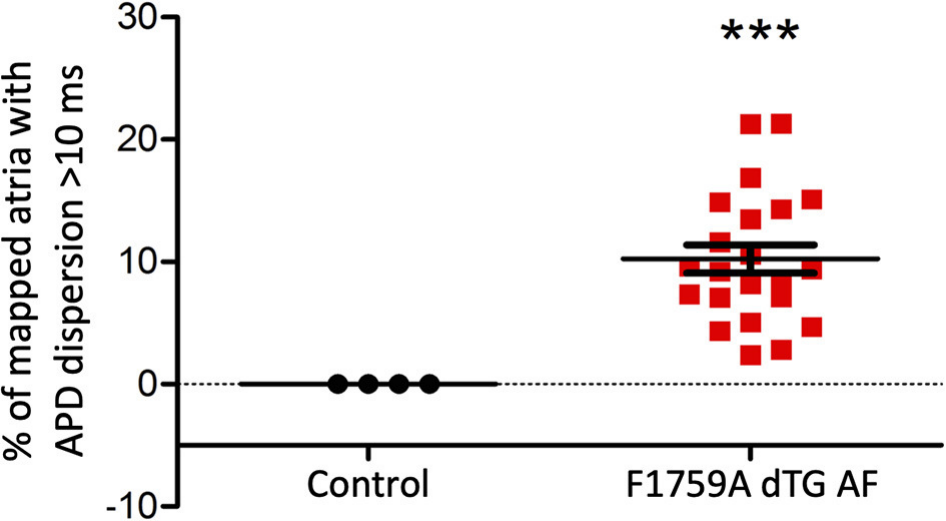
Analysis of the APD maps showing the percentage of areas with dispersion > 10 ms over the total epicardial area of the left atria imaged by optical mapping, in TG mice with AF. One sample *t*-test *p* < 0.05, *n* = 4 control and 22 TG mice. The average area of TG atrial tissue with dispersion > 10 ms was 10.24 ± 1.15%, while the control mice group with no AF was significantly different (***p < 0.001, Student's *t*-test) and did not exhibit any dispersion.

**Figure 5:**
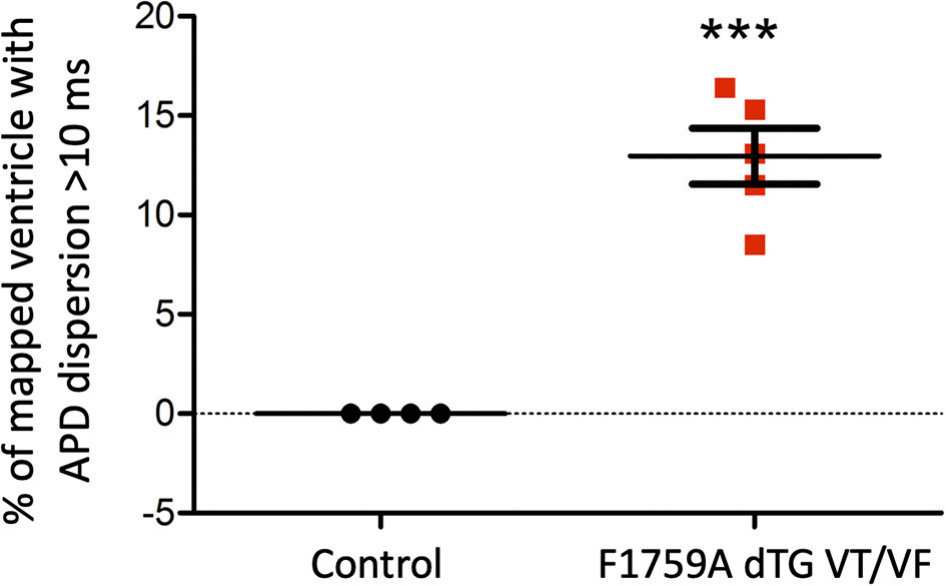
Analysis of the APD maps showing the percentage of areas with dispersion > 10 ms over the total epicardial area of the ventricle imaged by optical mapping, in TG mice with spontaneous VT/VF. One sample *t*-test *p* < 0.05, *n* = 4 control and 5 TG mice. The average area of TG ventricular tissue with dispersion > 10 ms was 12.9 ± 1.4%, while the control mice that exhibited no VT/VF were significantly different (***p < 0.001, Student's *t*-test) and did not show any dispersion.

We performed 2D epicardial surface voltage optical mapping on the atria and ventricles of Langendorff perfused TG mice (Figures 2, 3). Surprisingly, we observed islands or regions of APD dispersion patterns most consistent with the clumped phenotype in most of the hearts. We also analyzed the percentage of atrial and ventricular tissue imaged that exhibited these islands/regions by calculating the yellow (APD dispersion > 10 ms) to green area (APD dispersion < 10 ms) ratio on the binarized dispersion maps (Figures 2, 3, right column). In other terms, we measured the percentage of mapped tissue exhibiting APD dispersion > 10 ms (Figures 4, 5). We have previously shown that areas of reentry were most common in regions with APD dispersion > 10 ms (18). Hence, we used this cut-off to quantify the percentage of tissue with dispersion > 10 ms necessary for AF and VT/VF arrhythmogenesis, respectively.

Representative images from 3 TG AF mice mapped after hyperkalemic induced sinus conversion and 10 Hz pacing are displayed in Figure 2. These three reconstructions exhibit the clustered APD dispersion pattern, namely patchy islands of increased dispersion, especially near the atrial edges. This was found to be the predominant pattern of distribution in the left atrium for the 22 TG mice that were analyzed. The optical maps of the TG mice were compared to control mice in normal sinus rhythm without late persistent Na^+^ current, in which we had previously shown homogenous atrial APD maps (16). We observed in the three rows of representative images (Figures 2A–C) that there was heterogeneity of the APD maps, which did not appear to relate to anatomical structure, and the dispersion maps took the form of serpiginous patterns within the atria. We found that the islands/regions of APD dispersion might be singular, or that there might be an archipelago of regions within the atria. The percentage of areas with dispersion >10 ms over the total epicardial area of the atrial tissue imaged by optical mapping ranged from ~2 to ~22% (Figure 4). The average area of TG atrial tissue with dispersion > 10 ms was 10.24 ± 1.15%, while the control mice group with no AF was significantly different (*p* < 0.001, Student's *t*-test) and did not exhibit any dispersion, as seen on Figure 4.

As for the ventricular optical mapping results, serial representative images from three mice with spontaneous and sustained VT/VF that were captured in normal sinus rhythm are displayed in Figure 3. These three representative dispersion maps show clustered areas with mostly serpiginous patterns of APD dispersion. This was the predominant pattern of distribution in the ventricles of the five F1759A-dTG mice that were analyzed. The optical maps of the TG mice were compared to control mice in normal sinus rhythm without late persistent Na^+^ current, that had completely homogenous ventricular APD maps (16). The three rows of representative images (Figures 3A–C) show that there was heterogeneity of the ventricular APD maps. Just as in the atria, these areas of dispersion were inconsistent from mouse-to-mouse and did not appear to be correlated with anatomical ventricular structures, as the APD dispersion patterns were inconsistent from mouse to mouse. We found that the islands/regions of APD dispersion in the ventricle were similar to the patterns observed in atria. There were evident serpiginous islands/regions, consistent with the clustered phenotype. The percentage of areas with dispersion > 10 ms over the total epicardial area of the ventricle imaged by optical mapping ranged from ~8 to ~16% of the two-dimensional area (Figure 5). The average area of TG ventricular tissue with dispersion > 10 ms was 12.9 ± 1.4%, while the control mice that exhibited no VT/VF were significantly different (*p* < 0.001, Student's *t*-test) and did not show any dispersion (Figure 5).

## Discussion

There have been studies on dispersion as the substrate for persistence of arrhythmias like AF and VT/VF. Tissue spatial dispersion differences may be due to inherent alterations in cellular electrophysiological properties between cells. It may also be due to the presence of collagenous connective tissue septa, which may separate and insulate different myocardial muscle bundles to create a nidus for local conduction inhomogeneity (9, 19). Nevertheless, the methods to measure and quantify dispersion vary. Numerous methods, including measuring spatiotemporal electrogram dispersion, and the use of 12-lead ECG to determine the QT interval dispersion, have limited efficacy and correlate poorly with basic cellular electrophysiological data. Intracellular microelectrode recordings can be used to measure APD but has limited application, since it can be technically challenging to maintain stable cell membrane punctures. In addition, isolation of cardiac myocytes from ventricular tissue after enzymatic digestion can lead to various ventricular regional cardiomyocytes being measured at the same time (8). Other noninvasive clinical measurements of dispersion, such as 12-lead ECG QT dispersion, measure ventricular repolarization time and have been used for arrhythmia risk stratification. However, previous research studies have shown their limited ability to correlate dispersion with MAPs, as well as epicardial activation-recovery (8, 20).

To address these limitations, we used optical mapping of epicardial tissue recordings and applied it to the atria and ventricles of F1759A SCN5A TG mice in this study. This method relies on voltage sensitive dye fluorescence, captured by CMOS cameras, to obtain thousands of simultaneous recordings in both the atria and ventricles. The technique was used to study spatial APD dispersion in our TG mouse model with heterogenous cardiomyocyte increased late Na^+^ current, which allowed spontaneous and sustained AF and VT/VF (16). We presented theoretical models for APD dispersion, methods for analysis and calculation of APD dispersion, and showed that APD dispersion in clustered patterns is the predominant configuration for spontaneous occurrence and persistence of AF and VF in this murine model. We suggest herein that the presence of and quantification of APD dispersion of a tissue or organ may be a method to risk-stratify its propensity to propagate reentrant arrhythmia.

In the clinical electrophysiology laboratory, there are several methods for measuring tissue electrophysiological properties and refractoriness. Specific action potential tissue recordings can be obtained using a MAP catheter. However, MAP catheter electrograms may be distorted due to tissue movement, and maintaining tissue stability can be technically difficult. Electrophysiological measurement of effective refractory period can also be measured by transmitting regular trains of pacing stimuli followed by premature stimuli at progressively shorter coupling intervals. These measurements are limited to the tissue surface in contact with the catheter at epicardial or endocardial sites. However, consecutive measurements are required to estimate spatial dispersion (8, 15). Recently, a splined, multi-electrode catheter was used to record electrograms in the human atria in order to visualize electrogram dispersion sites, and simultaneously target these areas for ablation (13).

### Reentry as the Mechanism for Cardiac Arrhythmogenesis in the Atria and Ventricles

#### Atrial Dispersion

APD heterogeneity in the atria of the heart may be due to many factors, including age, gender, and pressure or volume overload, leading to increased fibrosis (21). Increased atrial fibrosis in patients with AF has been thought to be a nidus for reentry. Previous studies have suggested that fibrosis, no matter whether it is focal or diffuse in the atrial tissue, may act as a substrate for AF (22). 2D computational simulations suggest that fibrosis can lengthen APD, increase APD dispersion, and increase the likelihood of reentry (22, 23). Simulated fibrosis at small scales was associated with increased vulnerability to sustained reentry relative to fibrosis at larger scales. Other predisposition factors to AF include among other things altered ion channels expression, such as heterogeneous distribution of late sodium current (24). Besides, vagal stimulation shortens the atrial effective refractory period and APD, thus enhancing APD dispersion (25). Atrial dilation and remodeling may also be associated with an increased risk of AF inducibility (26, 27).

#### Ventricular Dispersion

The ventricular myocardium is thicker than the atrial myocardium and is more electrically heterogeneous, as it is comprised of at least three electrophysiologically and functionally distinct cell types: epicardial, M, and endocardial cells. These three principal ventricular myocardial cell types differ with respect to phase 1 and phase 3 repolarization characteristics (28). These differences in the three ventricular layers account for the transmural dispersion of repolarization, thus providing a substrate for the development of ventricular reentrant arrhythmias such as VT and VF. Ventricular arrhythmias, including VT/VF, can occur due to acute ischemia or infarction. They can also be seen in hearts with extensive cardiac remodeling, which may be caused by myocardial infarction, or by increased volume or pressure overload (14). Myocardial ischemia due to coronary artery disease has been associated with alterations in action potential duration. Downstream effects of ischemia, simulated by carbonyl cyanide-p-trifluoromethoxyphenylhydrazone (FCCP)-induced oxidative phosphorylation, cause an initial prolongation and then a subsequent reduction in APD. Action potential prolongation can be reduced by blocking the inward calcium current (*I*_Ca_), inward potassium current (*I*_K1_) and transient outward potassium current (*I*_to_). Alterations in *I*_Ca_, *I*_to_ and *I*_K1_ can be modulated by intracellular decreased ATP and pH or decreased calcium, all of which are metabolic fluctuations observed in ischemia (29).

Our TG F1759A SCN5A mouse model had episodes of spontaneous and sustained VT/VF, likely due to increased persistent late Na^+^ current, with modest reduction in ejection fraction. This model is not representative of all possible models of VT/VF as there are models of ventricular arrythmias due to structural heart disease such as myocardial infarction, ischemia or other ion channel mutations. However, we used this model herein to understand the possible APD dispersion pattern for reentry in VT/VF.

### Summary of Findings

Although three theoretical APD dispersion patterns were possible: uniform, random and clustered, the clustered pattern was the one overwhelmingly present in this mouse model. Underlying pathophysiological mechanisms that may cause clustered dispersion patterns rather than uniform or random in the atria and ventricles include fibrosis, genetic abnormalities or possibly infiltrative heart disease.

When we analyzed our optical mapping images for dispersion > 10 ms, APD dispersion patterns in both the atria and ventricles most often had one or more islands or regions within the tissue in either an ovoid or serpiginous shape. When measuring the percentage of areas with APD dispersion > 10 ms in the atria and ventricles, we found that commonly 10% of the overall tissue fell within the dispersion > 10 ms category.

Additionally, we found that the clustered patterns were likely not due to fibrosis, since histologically, there was only a small increase in fibrosis in AF TG mice compared to controls (16, 18). Our mouse model had only modest increase in fibrosis, but we acknowledge it may not be representative of all possible AF models. However, we used this model as a first step in understanding the possible APD dispersion patterns necessary for inducing reentry in AF.

Lastly, translation of our murine model findings to humans is critical. Optical mapping which uses blebbistatin, a calcium uncoupler, cannot be performed in patients *in vivo*. However, correlative findings may be obtained using MAPs and 3D electroanatomical mapping to locate and quantify areas of heterogenous conduction. Initial *in vivo* comparison between the mouse model and 5 patients undergoing AF ablation was previously performed by our group (18). Electroanatomical voltage maps and MAP recordings in sinus rhythm for these patients successfully found APD heterogeneity, similarly to APD dispersion in mice. In the advent of new technologies and in the era of high-density mapping, we envision the development of new high density catheters allowing measurement of APDs at high resolution at several myocardial tissue locations simultaneously. This information may be used to further stratify patients at risk in addition to other standard clinical diagnostic tests.

### Limitations and Future Work

A major limitation of standard 2D optical mapping is its inability to view the structure in three dimensions. Hence, the appearance of clustered patterns near the edge of the tissue border might actually be due to the abrupt curvature of the tissue that could not be appreciated in two-dimensional imaging. Our group has since then developed a three-dimensional panoramic optical imaging set-up, currently being investigated in other mouse models, that could help overcome this limitation in future studies.

Moreover, we are developing a 3D visualization tool to co-register multiple imaging modalities for improved computational modeling and risk stratification of arrhythmias. On top of tissue electrophysiological information from panoramic optical imaging such as activation mapping and APD measurements, we have integrated and overlayed the data and structural information extracted automatically from anatomical imaging techniques such as computed tomography (CT) or magnetic resonance imaging (MRI) with deep learning algorithms. The purpose of this integration would be to study the effects of structural changes such as fibrosis and hypertrophy on arrhythmogenesis. These measurements can be derived through myocardial wall thickness measurements from the reconstructed CT, while MRI scans can give us access to complementary information like fibrosis and myocardial fiber orientation. Merging the spatial information from different imaging modalities may allow for a more in-depth three-dimensional characterization of the arrhythmogenic substrate. Visualizing how anatomical markers correlate with arrhythmia mechanisms and translating this information clinically would result in improved understanding of disease progression. Consequently, interactions and inter-dependence of functional and structural measurements through the fusion of multi-imaging modalities may lead to better arrhythmia risk stratification performance.

Finally, our study is limited by the usage of a single mouse model but it is an *in vivo* model with known spontaneous and sustained atrial and ventricular arrhythmias, and exhibits structural and functional changes similar to what is observed in human patients with these clinical arrhythmias. Future investigation will include additional pacing experiments in this murine model to confirm that tissues with areas of high APD dispersion have a high likelihood of inducible reentrant arrhythmias. Further studies evaluating the arrhythmia risk stratification performance of the APD dispersion computational analysis approach presented herein may provide further insight into how to study, prevent and treat patients.

## Conclusion

Analysis and discussion of APD dispersion patterns, which may be a leading cause of cardiac arrhythmogenesis, may be helpful to improve computational modeling and justify further studies to characterize and quantify APD dispersion for risk stratification of atrial and ventricular arrhythmias. Further comprehension of dispersion patterns may lead to better understanding of whether and how reduction of APD dispersion may lead to new treatment strategies for patients with arrhythmias.

## Data Availability Statement

The raw data supporting the conclusions of this article will be made available by the authors, without undue reservation.

## Ethics Statement

The animal study was reviewed and approved by The Institutional Animal Care and Use Committee at Columbia University.

## Author Contributions

UA and EW designed the study, performed experiments, and collected and analyzed the data. UA, LM, JK, SL, and EW wrote the manuscript. UA and LM were involved in generating the figures. All authors contributed to the article and approved the submitted version.

## Conflict of Interest

The authors declare that the research was conducted in the absence of any commercial or financial relationships that could be construed as a potential conflict of interest. The handling editor declared a past collaboration with the author EW.
